# Reconsideration of the lifetime limit of 50 rebated TMS sessions

**DOI:** 10.1177/10398562261431257

**Published:** 2026-02-26

**Authors:** Saxby Pridmore, Yvonne Turnier-Shea, Marzena Rybak, Gregory M. Peterson

**Affiliations:** Discipline of Psychiatry, 3925 University of Tasmania, Hobart, TAS, Australia; Psychiatrist, Hobart TMS, Bellerive, TAS, Australia; School of Pharmacy and Pharmacology, 3925 University of Tasmania, Hobart, TAS, Australia

**Keywords:** transcranial magnetic stimulation, treatment resistant depression, major depressive disorder

## Abstract

**Objective:**

To make a case for the Medicare benefits schedule (MBS) rule, that depressed patients who benefit from transcranial magnetic stimulation (TMS) have a lifetime limit of 50 treatments, to be reconsidered.

**Conclusion:**

Evidence proves TMS can produce remission in major depressive disorder/treatment resistant disorder (MDD/TRD) when medication has failed. This is a relapsing disorder, and further treatments are usually required (but are prevented by this limit). We suggest the limit be raised to 70 treatments (2 courses, or similar) per year.

In 1957, imipramine was identified as the first medication to have an antidepressive action. Subsequently, various waves of newer antidepressants have made modest improvements in effectiveness. More recently psychiatry has admitted a range of procedures which have valuable antidepressant effects—one of these is transcranial magnetic stimulation (TMS).

We make the case that the Medicare benefits schedule (MBS) rule that those patients who benefit from transcranial magnetic stimulation (TMS) treatments can receive rebates for only 50 treatments throughout their lives is unreasonable. We make the observation that other patients have access to 50 rebated for one-hour consultations with a psychiatrist per year, for an unlimited number of years. In fairness to those who benefit from therapeutic TMS, the lifetime limit of 50 treatments should be replaced with an allowance of 70 treatments (equivalent of two courses) per year.

Major Depressive Disorder (MDD) is a common, severe, chronic disorder—the lifetime prevalence is 10–15%,^1^ and 3.5% of depressive patients die by suicide. In addition to patient suffering, the economic costs to society of MDD are great—involving both medical care and loss of productivity—in 2019, the annual cost to the USA was US$333.7 billion.^2^ There is great interest in reducing this impost.

Only 37% of MDD patients achieve remission with a first course of antidepressant medication, and 31% with a second.^3^ MDD which does not respond to two courses of different antidepressant medication has been termed treatment resistant depression (TRD). The Australian MBS has determined—when TRD status is deserved, a course (35 treatments) of TMS is appropriate and may be rebated—and some months later, if further treatment is required, an additional short course of 15 sessions, exhausts the lifetime allowance.

MDD has a high relapse rate,^4^ and the more difficult it is to bring an acute episode to remission, the more likely is an early relapse—thus, TRD is very prone to relapse.^5^ On average, depressive patients experience 0.30 depressive episodes per year.^6^

In MDD, TMS treatment has three different applications. The first and second are similar, the first being the initial treatment course which is provided to a patient experiencing acute TRD. Except in very severe depression displaying psychotic, melancholic, catatonic or suicidal features, which indicate ECT should be immediately considered, TMS is the next logical treatment, when medication has failed. Evidence indicates, in real world acute TRD, TMS will provide remission in 28–62% of cases.^7^

The second application of TMS occurs when a TMS induced remission relapses and further treatment of acute depression is required. When an acute episode of MDD has been brought to remission with TMS, it is highly probable that another TMS treatment will bring another remission.^8^ Following TMS induced remission, 54% of patients will have relapsed at 12 months.^9^ Accordingly, TMS is often required for further acute treatment. [Similarly, post successful ECT, despite continuation treatment, approximately 50% of patients will relapse in the first year and require further treatment.^5^].

For initial acute and relapse TMS treatment of MDD/TRD, two leading protocols are effective. Low frequency stimulation (1 Hz) applied to the right dorsolateral prefrontal cortex and high-frequency stimulation (≥10 Hz) applied to the left dorsolateral prefrontal cortex. Depending on the form chosen a treatment takes 15–37.5 minutes and one treatment is provided each weekday for up to 7 weeks (total: 35 treatments). More recently, patterned stimulation (theta burst; TBS) has entered clinical practice. Depending on the form chosen, a single theta burst treatment takes 40 seconds to 3.5 minutes. Accelerated TBS refers to multiple treatments in 1 day, a form which is gaining popularity in many centers.

Third, “maintenance” TMS is treatment aimed at maintaining remissions. It takes two forms—“continuation TMS” and “cluster maintenance.” Continuation TMS: one treatment is delivered episodically. In the first report, one treatment was provided at weekly intervals for 1 year—with some encouraging results.^10^ In a randomized clinical trial of continuation TMS, remitted patients received a once weekly TMS treatment or lithium pharmacotherapy (an established mood stabilizer) for 24 weeks.^11^ There was no significant difference in relapse rates, indicating TMS provided stabilization equal to lithium. Various continuation protocols have been described—those using treatment intervals of greater than 2 weeks are less effective.

Cluster maintenance was developed in Australia—clusters of five treatments over 2–5 days are repeated at monthly or greater intervals.^12^ In an assessor blinded, randomized, controlled 12-month trial, the outcomes of three groups were compared. One group received clustered TMS alone, another received clustered TMS plus antidepressant medication, and the third received antidepressant medication alone. Both TMS containing groups experienced significantly lower relapses rates than the group receiving only antidepressant medication—indicating cluster TMS is superior to antidepressant medication in the prevention of relapse.^13^

The MBS does not accept the value of maintenance TMS and does not fund this form of maintenance. While not the main thrust of this Viewpoint, evidence of the value of maintenance TMS continues to grow,^14^ and funding of this form of treatment should be reconsidered.

Regular consultations with a clinical psychiatrist may contribute positively to patient mental health. In Australia, treatment provided by a psychiatrist may take the form of one (possibly more) rebated 50 minute consultations/treatments per week. The MBS rebates 50 such treatments per year. Should more than 50 treatments be required in 1 year, another item number, which carries eligibility criteria, makes this possible. There is no limit on the number of years for which this arrangement can apply.

In summary, it has been proven that (1) MDD/TRD is a common, severely disabling and distressing disorder,^1^ (2) a third of patients with MDD/TRD do not achieve remission with pharmacological treatment,^3^ (3) in real world studies, between 30 and 50% of those who have not achieved remission with pharmacological treatment will achieve remission with TMS,^7^ and (4) MDD/TRD is most commonly a relapsing disorder, and therefore, most patients will require, in addition to the initial treatment, further acute treatments over time.^8^

Accordingly, it is illogical/discriminatory to allow more than 50 rebated one-hour psychiatrist sessions per year (for any number of years) for appropriate services but not to allow more than 50 rebated TMS treatments in a lifetime for severe depression treatment. This decision may have originally been based in the mistaken belief that TMS is prohibitively expensive—in fact, more than 20 studies have found TMS to be more cost-effective than either medication or ECT treatment.

Our suggestion of an annual limit to 70 treatments is based on the facts that the accepted length of a course of acute TMS (for MDD) is 35 treatments and that relapse within a year is quite common. An allowance of 70 treatments would enable two complete treatments per year. Should the MBS come to recognize maintenance TMS, this increased annual number would enable one acute treatment and maintenance treatment for more than 6 months.

From January 1, 2026, the Department of Veteran’s Affairs has increased the number of allowed TMS treatments for TRD. The DVA is now allowing 100 treatments per year, and a lifetime limit of 300 sessions per patient. If the DVA position is more palatable to the MBS authorities, than our proposal, it would be a reasonable compromise—the MBS might move to the DVA position. This would allow a greater number of deserving patients to receive this safe and effective care for this painful and dangerous disorder than is currently the case.

## Data Availability

This is a statement of opinion and does not involve other data.*
